# Conducting polymer-based multilayer films for instructive biomaterial coatings

**DOI:** 10.4155/fso.15.79

**Published:** 2015-11-02

**Authors:** John G Hardy, Hetian Li, Jacqueline K Chow, Sydney A Geissler, Austin B McElroy, Lindsey Nguy, Derek S Hernandez, Christine E Schmidt

**Affiliations:** 1 J Crayton Pruitt Family Department of Biomedical Engineering, University of Florida, Gainesville, FL 32611, USA; 2 Department of Biomedical Engineering, The University of Texas at Austin, Austin, TX 78712, USA

**Keywords:** biomaterials, cellular alignment, conducting polymers

## Abstract

**Aim:**

To demonstrate the design, fabrication and testing of conformable conducting biomaterials that encourage cell alignment.

**Materials & methods:**

Thin conducting composite biomaterials based on multilayer films of poly(3.4-ethylenedioxythiophene) derivatives, chitosan and gelatin were prepared in a layer-by-layer fashion. Fibroblasts were observed with fluorescence microscopy and their alignment (relative to the dipping direction and direction of electrical current passed through the films) was determined using ImageJ.

**Results:**

Fibroblasts adhered to and proliferated on the films. Fibroblasts aligned with the dipping direction used during film preparation and this was enhanced by a DC current.

**Conclusion:**

We report the preparation of conducting polymer-based films that enhance the alignment of fibroblasts on their surface which is an important feature of a variety of tissues.

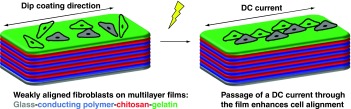

## Abstract (lay abstract)

**Lay abstract:** Cells inhabit environments known as the extracellular matrix (ECM) which consists of a mixture of different biomolecules, and the precise composition and topographical properties are different in different tissues (e.g., bone, brain, muscle, skin). Cells interact intimately with the ECM, not only constructing the biomolecules, but assist its organization in 3D space, and its degradation (which is important for tissue remodeling); reciprocally, cells respond to the ECM (e.g., by modifying their size, shape, etc). Cellular alignment is observed in organs and tissues such as bones, muscles and skin, and this alignment is important for the healthy functioning of the organ/tissue. Here, we present a novel method of aligning cells on biomaterials, simply by applying an electrical current through the biomaterial.

**Figure 1.**
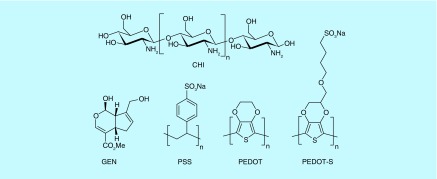
**Molecules employed in this study.** CHI: Chitosan; GEN: Genipin; PEDOT: Poly(3,4-ethylenedioxythiophene); PEDOT-S: Sulfonated PEDOT derivative; PSS: Polystyrenesulfonate.

**Figure 2.**
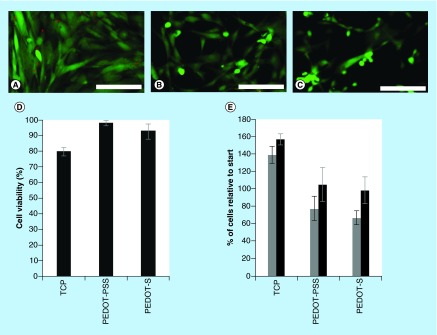
**Adhesion and growth of human dermal fibroblasts on various surfaces up to 4 days in culture.** **(A)** Tissue culture plate controls. **(B)** PEDOT-PSS-based multilayer films. **(C)** PEDOT-S-based multilayer films. Cells were stained with a LIVE/DEAD^®^ Viability/Cytotoxicity Kit, live cells were green and dead cells were red. Scale bars represent 250 µm. **(D)** Cell viability after 4 days in culture as determined with a LIVE/DEAD^®^ Viability/Cytotoxicity Kit. **(E)** Number of cells adhered to the substrates after 2 days (grey bars) or 4 days (black bars) as estimated by the AlamarBlue^®^ assay. Error bars represent standard deviations (n = 3).

**Figure 3.**
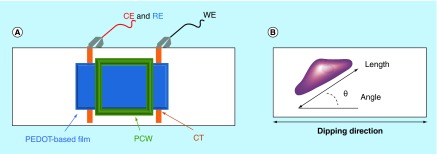
**(A) Experimental setup for electrical stimulation of PEDOT-based films (not to scale). (B) Cell alignment and length assessment.** CE: Counter electrode; CT: Copper tape; PCW: Polycarbonate well; RE: Reference electrode; WE: Working electrode. Adapted with permission from [33] © 2015 Hardy JG *et al*.

**Figure 4.**
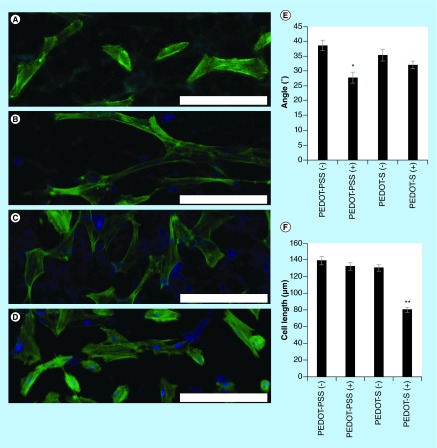
**Analysis of the morphology of human dermal fibroblasts on various surfaces with optional electrical stimulation (applied in line with the dipping direction and horizontally relative to the images presented).** **(A)** PEDOT-PSS-based multilayer films without electrical stimulation, PEDOT-PSS(-). **(B)** PEDOT-PSS-based multilayer films with electrical stimulation, PEDOT-PSS(+). **(C)** PEDOT-S-based multilayer films without electrical stimulation, PEDOT-S(-). **(D)** PEDOT-S-based multilayer films with electrical stimulation PEDOT-S(+). DAPI-stained nuclei are blue and Alexa Fluor^®^ 488-stained actin is green. Scale bars represent 200 µm. **(E)** Assessment of cell alignment. **(F)** Assessment of cell length. Error bars represent standard errors of the mean (n = 150 or more). *p ≤ 0.01, **p < 0.001.

The specific properties of tissues act as cues (individually or in concert) that determine the behavior of cells that inhabit them, and knowledge of these properties can be utilized to engineer instructional tissue scaffolds to facilitate the regeneration of functional tissues [1–6]. The topographical properties of tissues may instruct cells to align, as is clearly observable within the anisotropically aligned pores observed in bone, cardiac, nerve and other tissues [7–10]. Inspired by this, scientists and engineers have reported novel methodologies of imparting biomimetic topographical features to biomaterials [7–10].

Endogenous electric fields are another cue influencing cell behavior, including processes such as embryogenesis and wound healing. Electrical fields are also known to align cells, with endogenous electric fields playing a role in the alignment of cells during development of the nervous system [6], and exogenous electric fields have been shown to align a variety of cell types (including astrocytes, epithelial cells, fibroblasts) *in vitro* [6]. This motivates the development of biomimetic electrically conductive materials that instruct cell alignment aided by application of an electrical field/current that mimics endogenous electrical fields/currents [6,11].

Conducting biomaterials based on conducting polymers (CPs) such as derivatives of polyaniline, polypyrrole or polythiophene, have potential for both long-term biomedical applications (e.g., electrodes) and short-term biomedical applications (e.g., drug delivery or tissue engineering) [11–14]. CP-based scaffolds have been developed for the regeneration of bone and nerve tissues, and organs including the heart and skin [11–14].

Layer-by-layer assembly is a method of producing thin multilayer films that was popularized by Möhwald and others [15,16]. Conductive multilayer films have been prepared from a variety of organic electronic components, often for applications in energy, of which a number of examples based on CPs exist [17]. Wallace and co-workers reported the preparation of bioerodible CP-based multilayer films based on an anionic sulfonate-displaying polythiophene derivative (with a *M*_w_ = 13,160 Da and *M*_n =_ 6682 Da as determined by GPC) and cationic polyethyleneimine (17 kDa) [18], both of which are below the renal filtration limit of approximately 50 kDa [11], and the films were shown to be suitable for the growth and proliferation of cells derived from the connective and muscle tissue of mice (L929 and C2C12 cells, respectively) [18]. They subsequently showed that it was possible to prepare CP-based multilayer films based solely on anionic and cationic polythiophene derivatives that could be disassembled upon the application of an electrochemical trigger (a potential step of 650 mV for 19–42 h) [19]. Wei and co-workers reported the first example of biologically functional biodegradable CP-based multilayer films for bone tissue engineering, that were used for the electrical stimulation of osteoblast precursor MC3T3-E1 cells (derived from mice) that resulted in increased expression of osteopontin and runt-related transcription factor 2 which are markers of osteogenesis [20].

Here we describe the preparation of CP-based films composed of poly(3,4-ethylenedioxythiophene) (PEDOT) derivatives, chitosan and gelatin that are cross-linked by genipin (Figure 1). These conducting films enabled the electrical stimulation of human dermal fibroblasts cultured thereon which resulted in the preferential alignment of the cells with a DC current passed through the films, which is the first report of such a phenomenon on a conducting polymer-based material.

## Materials & methods

Unless otherwise stated, all chemicals were of ACS grade, purchased from Sigma-Aldrich and used as received without further purification, for example, poly(3,4-ethylenedioxythiophene)-poly(styrenesulfonate) 1.1% in H_2_O, neutral pH, high-conductivity grade (PEDOT-PSS), chitosan (70 kDa), phosphate buffered saline (PBS) tablets, etc. Hydroxymethyl EDOT was purchased from Sarchem Laboratories, Inc., and ITO slides were purchased from Ted Pella, Inc. For cell culture experiments, all reagents were purchased from Invitrogen (CA, USA) unless otherwise stated. Human dermal fibroblasts (HDFs) were purchased from Lonza (MD, USA).

## Film preparation

Aminopropyltrimethoxysilane-functionalized glass microscope slides were used as positively charged substrates for the deposition of conducting polymer-based films [21]. Negatively charged polymers were either poly(3,4-ethylenedioxythiophene)-poly(styrenesulfonate) 1.1% in H_2_O, neutral pH, high-conductivity grade (PEDOT-PSS), or PEDOT-S; whereas the positively charged polymer was chitosan (70 kDa) that was dissolved in aqueous acetic acid (1% v/v).

Drop cast films were prepared by briefly vortexing aqueous solutions of the polymers (typically 1 mg ml^-1^, adjusted to pH 7 and buffered with 10 mmol PBS), casting on glass slides, air drying for 24 h and then drying under vacuum for 48 h.

Multilayer films were prepared from aqueous solutions of the polymers (typically 1 mg ml^-1^, adjusted to pH 7 and buffered with 10 mmol PBS) using a Gilson 223 Sample Changer (Gilson, Inc., WI, USA) converted for use as a dip coater controlled by a script written in LabVIEW (National Instruments, Inc., TX, USA) which is available in the Supplementary Information. Films were deposited by repetitive sequences of: dipping in negatively charged polymer solution (dip time 15 min), air drying 1 min, rinsing in water (dip time 30 s), air drying 1 min, dipping in chitosan (70 kDa, dip time 15 min), air drying 1 min, rinsing in water (dip time 30 seconds), air drying 1 min. The films were air dried for 24 h and then dipped in a solution of gelatin (1 mg/ml, dip time 15 min), air dried for 1 min, rinsed in water (dip time 30 s), air dried 1 min, rinsed in water (dip time 30 s), air dried for 24 h and vacuum dried for 48 h.

### Profilometry

Profilometry was carried out using a Veeco Dektak 6M Stylus Profilometer (Veeco Instruments Inc., NY, USA) fitted with a 12.5 μm stylus tip. The profilometer was isolated on an air table to reduce ambient vibrations. The profilometer was operated at 10 mg of stylus force, and used to record profiles of distances of ca. 1 cm, recording data points every 555 nm. Data analysis was carried out with the software provided by the manufacturer, which allowed the determination of the thickness and roughness of the films. The surface roughness parameters are analyzed and reported in accordance with the ISO 25178 series. The average roughness (*Ra*) is the arithmetic average of the deviation from the mean line, and is the most used international parameter of roughness, and the root-mean-square roughness (*Rq*) is based upon this.

## Water contact angle measurements

Measurements were carried out with a high-speed contact angle measurement device (FTA200 video-based semiautomatic contact angle goniometer supplied by First Ten Ångstroms, Inc., VA, USA). Images of a drop of deionized water (2 µl) laid on the surface of the samples were recorded at a frame rate of 360 frames per second, and the contact angles for the droplets were recorded after 3 s of contact with the film. The reported values are the average of at least three measurements at different positions on a film.

## Conductivity determination

Resistance (*R* in Ω) was measured between two silver electrodes using a digital multimeter (DM-8A, Sperry Instrument, WI, USA). The resistivity, ρ (Ω/cm), of the films was determined in accordance with Equation 1:
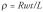



The resistance, *R*, was recorded in at least ten different positions on the materials, *W* is the width of the film (typically 2.5 cm), *t* corresponds to the thickness of the film (as determined via profilometry) and *L* is the distance between the two silver electrodes (typically 0.5 cm). The conductivity (S/cm) of the films was determined in accordance with Equation 2:
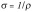



### Sample preparation & conditions for *in vitro* cell culture

Samples were sterilized by incubation in 70% ethanol solution, followed by exposure to UV for 30 min. After sterilization, films were incubated for 30 min under 3 mm of HDF growth medium composed of: high glucose Dulbecco's modified Eagle medium (DMEM, 440 ml); fetal bovine serum (50 ml); antibiotic–antimycotic (5 ml); non-essential amino acids (5 ml) and 2 ng ml^-1^ basic fibroblast growth factor. Medium was aspirated and replaced prior to HDF seeding. Cell viability before starting the experiment was determined by the Trypan Blue (Sigma, USA) exclusion method, and the measured viability exceeded 95% in all cases. Cells were seeded at 5000 cells per cm^2^ under 3 mm of medium, and incubated at 37°C, 95% humidity and a CO_2_ content of 5%.

### Cell proliferation studies

After 2 days the cells were washed gently with PBS, followed by the addition of fresh medium containing 10% v/v of the AlamarBlue^®^ reagent. After 2.5 h of culture, the medium was aspirated and replaced with fresh medium, and 100 µl of the aspirated medium containing the AlamarBlue reagent was placed in a 96-well plate, and the fluorescence was measured with a fluorimeter (Synergy HT Multi-Mode Microplate Reader, Biotek US, Winooski, VT). Two controls were considered during the measurement of the fluorescence: the first was wells containing medium alone (i.e., no cells or AlamarBlue reagent), which was not fluorescent; and the second was wells that contained the AlamarBlue reagent but no cells (used for baseline correction). Numbers of cell adhered to the various surfaces studied herein are reported relative to their initial seeding density of 5000 cells per cm^2^, which was assigned an arbitrary value of 100%. After another 2 days (i.e., at 4 days after initial seeding) this process was repeated. The medium was aspirated and replaced once more at 6 days after initial seeding, and finally after a total of 8 days in culture the viability of the cells was evaluated using a LIVE/DEAD^®^ Viability/Cytotoxicity Kit for mammalian cells (Molecular Probes, Eugene, OR). The medium was removed and cells on the surfaces were incubated with 4 µM ethidium and 2 µM calcein AM in PBS for 15 min at 37°C in the dark. Live cells were stained green because of the cytoplasmic esterase activity, which results in reduction of calcein AM into fluorescent calcein, and dead cells were stained red by ethidium, which enters the cells via damaged cell membranes and becomes integrated into the DNA strands. Fluorescence images of cells were captured using a color CCD camera (Optronics^®^ MagnaFire, Goleta, CA, USA) attached to a fluorescence microscope (IX-70; Olympus America Inc.). Cells were counted with the cell counter tool (plugin) in the open source program ImageJ, all cells on all images were counted. Results of AlamarBlue assays presented are the average of four samples and ethidium/calcein stained images are representative of three samples (typically three images per sample).

### Cell orientation studies in the absence or presence of electrical stimulation

Cell orientation studies employed a custom built setup. Electroactive multilayer films supported on glass slides were (width of 2.5 cm, length of 7.5 cm) were sterilized by incubation in 70% ethanol solution, followed by exposure to UV for 30 min. Polycarbonate wells (square polycarbonate blocks, thickness of 1 cm, sides of 2.5 cm, with square holes with sides of 0.9 cm cut out), Dow Corning^®^ high vacuum grease, and medium binder clips (Staples^®^, MA, USA) were sterilized by autoclaving. Holes were drilled into the sides of 10-cm polystyrene Petri dishes using a Dremel saw (Lowes, Mooresfield, NC, USA), and the plates were sterilized by exposure to UV for 60 min. Adhesive-backed copper tape (5-mm width, Ted Pella, Inc.), waterproof Kapton^®^ tape (1-cm width, Fisher Scientific, MA, USA), wires and alligator clips were sterilized by exposure to UV for 60 min.

Electroactive multilayer films supported on glass slides and secured in position with two thin strips of adhesive-backed copper tape that were attached to the films, parallel to one another and separated by a distance of ca. 4 cm. One face of the polycarbonate wells was coated with vacuum grease and placed on the electroactive tissue scaffolds, greased side down, in contact with the glass slide. A binder clip on either side of the well was used to secure this in position and render it water tight. A strip of copper tape was run between the parallel copper strips attached to the scaffolds and the ends of the slides as points of contact for the alligator clip-terminated wires attached to the multipotentiostat (CH Instruments, TX, USA). The counter and reference electrodes were connected together and clipped to copper tape on one side of the slide, and the working electrode was clipped to copper tape on the other side of the slide. HDFs were plated and cultured for 1 day as described above. A potential step of +10 mV mm^-1^ was placed across the substrate for the duration of 4 h, after which the wires were disconnected and the substrates cultured as normal for a further 40 h. The medium was aspirated and the samples were washed gently with PBS. Cells were fixed with 4% paraformaldehyde in PBS for 15 min, permeabilized with 0.1% Triton X-100 (Fluka) and 2% bovine serum albumin (BSA) in PBS buffer for 5 min, followed by blocking with 2% BSA in PBS buffer for 30 min at room temperature. Actin filaments and cell nuclei within cells were stained with Alexa Fluor 488^®^ Phalloidin (Life Technologies, USA) for 30 min and 4′,6-diamidino-2-phenylindole (DAPI, Invitrogen, USA) for 5 min. The cells were then washed three times with PBS buffer and stored at 4°C until images were acquired. Fluorescence images of cells were captured using a color CCD camera (Optronics^®^ MagnaFire, CA, USA) attached to a fluorescence microscope (IX-70; Olympus America Inc.). Images are representative of three samples. Images were analyzed using ImageJ. A line was drawn across cells for measurements, and lengths in pixels were converted to lengths in µm using the measure bar in ImageJ. Angles were determined measuring from the left side of the image to the right side of the image to ensure that all angles measured would be within the 1st and 2nd quadrants. In Excel the absolute value of the negative angles were taken so all angles measured would be converted to the 1st quadrant, and these data were converted to cartesian coordinates. Scatter plots were generated automatically using a polar plot add-in [22] and the data transferred to Excel for further calculations. A minimum of 150 cells were counted per experiment, and error bars represent standard errors.

## Statistics

To assess cell behavior on the materials, a one-way ANOVA was performed using SPSS Statistics 22. Tukey *post hoc* tests were used to determine significance between groups, denoted *p ≤ 0.01, **p < 0.001.

## Results & discussion

Films were deposited on nonconductive glass slides that were previously rendered positively charged by modification with aminopropyltrimethoxysilane. The polyelectrolyte complex composed of PEDOT and polystyrenesulfonate (PSS), PEDOT-PSS, or the sulfonated PEDOT derivative PEDOT-S (Figure 1) were used for film preparation. They are both electrochemically stable over periods long enough to facilitate short-term electrical stimulation of tissues in their vicinity [23] and have been shown to be relatively nonimmunogenic after implantation in various tissues in mice and rats for periods of several weeks [11]. The polysaccharide chitosan was chosen as the cationic polymer to interact with the anionic PEDOTs (PEDOT-PSS and PEDOT-S, respectively) as it is also known to be relatively nonimmunogenic (particularly by comparison with polyethyleneimine) [24,25]. Furthermore, electrochemically reducing the backbone of PEDOT-S makes it less positively charged, which in turn reduces the number of sulfonates necessary to dope the polymer backbone rendering the surface charge of PEDOT-S films to be predominantly negative, therefore, PEDOT-S can be employed as a surface coating that enables electrochemically triggered cell desorption [26]. Therefore we employed gelatin as a surface coating to render the films cell adhesive, and cross-linked the films with genipin (a natural cross-linker of biopolymers including chitosan, collagen and gelatin, which is markedly less toxic than the more commonly used glutaraldehyde) [27–30], to ensure film stability for the duration of the experiments.

Film preparation by drop casting yielded films with μm-scale roughnesses, water contact angles (WCAs) of approximately 50° and conductivities of the order of 10^-7^ S cm^-1^ (Table 1). By contrast, layer-by-layer assembly via dip-coating (Supplementary Figure 1) yielded multilayer films that were smoother (nm-scale roughnesses) and more conductive, 10^-4^ to 10^-3^ S cm^-1^ (Table 1), that are on a similar order of magnitude to those of mammalian tissues (typically ≥ 10^-4^ S cm^-1^) [31].

With a view to the application of the multilayer films as coatings for biomaterials we cultured human dermal fibroblasts (HDFs) on their surfaces and compared them to commercially available tissue-culture treated Corning^®^ Costar^®^ tissue culture plate (TCP) controls. HDFs cultured on TCP controls adhered to and spread on the TCP as expected (Figure 2A). HDFs had somewhat more rounded morphologies when cultured on the PEDOT-PSS-based or PEDOT-S-based multilayer films (Figure 2B & C) which is indicative of slightly poorer cell adhesion to the conducting substrates despite the gelatin coating. We believe that this is the result of differing surface chemistry of the substrates. Indeed, the conducting polymers have a net negative charge which will be moderately repellent towards negatively charged cells. Moreover, polystyrene cell culture plates are more hydrophobic (water contact angle 98.3° ± 3.7°) [32] than the negatively charged conducting polymer substrates, and analogous differences in water contact angle have been shown to affect the adsorption of cell adhesive proteins to the substrates, leading to poorer cell adhesion on the negatively charged substrates. Interestingly, we found that cell viability for the HDFs cultured on the PEDOT-based films was comparable, and indeed somewhat better than for HDFs cultured on TCP controls (Figure 2D). We found that HDFs adhered to and proliferated on the surface of all of the substrates over the period of 4 days. HDFs cultured on the TCP control substrates proliferated somewhat faster than on the PEDOT-PSS-based or PEDOT-S-based films (Figure 2E), which is similar to the findings of Wallace and co-workers with mouse derived L929 and C2C12 cells on polythiophene-based films [18].

To assess the potential of the PEDOT-based films to act as instructive coatings for biomaterials, we investigated four different systems: cells seeded on PEDOT-PSS-based multilayer films without electrical stimulation; cells seeded on PEDOT-PSS-based multilayer films with electrical stimulation; cells seeded on PEDOT-S-based multilayer films without electrical stimulation; and cells seeded on PEDOT-S-based multilayer films with electrical stimulation using a custom built setup (Figure 3A). Those samples without electrical stimulation were cultured for 3 days, whereas those that were electrically stimulated were cultured for 1 day without stimulation, followed by one period of stimulation at 10 mV mm^−1^ for 4 h, then 44 h without stimulation.

We found that both the PEDOT-PSS-based and PEDOT-S-based substrates were stable to electrical stimulation at 10 mV mm^-1^ for 4 h, and moreover that HDFs maintained adherence to the substrates after electrical stimulation. We observed that HDFs displayed a moderate preference for aligning with the direction in which the slides were dipped during the dip coating process (Figures 3B & 4). Cells without any preferential alignment would be expected to have an average orientation of 45° (Figures 3B & 4 & Supplementary Figure 2); however, we saw that HDFs on PEDOT-PSS-based multilayer films had an average orientation of 38.6° ± 1.5° (Figure 4A & E), whereas HDFs on PEDOT-S-based multilayer films had an average orientation of 35.3° ± 1.8° (Figure 4B & E), which is likely to be because the polymer chains were aligned during the dip coating process as is commonly observed for multilayer films prepared in this fashion [34], and fibroblasts are known to respond to features on such length scales [35,36]. Interestingly, electrical stimulation of HDFs on the conductive substrates led to an increase in their propensity to align with the direction of the DC current passed through the substrate (akin to their behavior when a DC current is passed through the culture medium) [37], and we found that HDFs on PEDOT-PSS-based multilayer films had an average orientation of 27.7° ± 1.7° (Figure 4C & E), whereas HDFs on PEDOT-S-based multilayer films had an average orientation of 32.1° ± 1.1° (Figure 4C & E). The fact that the increase in cell alignment was somewhat lower for the cells on the PEDOT-S-based multilayer films than the PEDOT-PSS-based multilayer films is probably because of changes in the surface chemistry of the films that alter cell-substrate interactions as reported by Berggren and co-workers for PEDOT-S-based films [26]. Further evidence in support of this hypothesis can be found in changes in the length of the cells on the PEDOT-S-based films after electrical stimulation, with their average lengths reduced from approximately 135–80 μm (Figures 4C, 4D, 4F & Supplementary Figure 2). Indeed, passing a current through the conducting polymers alters the charge upon them (in this case, making it less positively charged) which leads to reduced numbers of negatively charged sulfate groups interacting with the positively charged backbone of the conducting polymer, and therefore the surface of the films is somewhat more negatively charged. Alterations to the surface chemistry of the substrates affects the adhesion of both biomolecules (e.g., proteins, glycoproteins and polysaccharides) and cells to the surface. Logically, negatively charged cells interact with negatively charged substrates relatively weakly, an effect employed by others to engineer surface coatings enabling electrochemically triggered cell desorption [26]. Nevertheless, the moderately increased cell alignment on both PEDOT-PSS-based and PEDOT-S-based multilayer films suggests that they may find application as instructive biomaterial coatings, potentially for tissue engineering of skin or a variety of other niches (such as bones, muscles, nerves, skin and tendons) in which cell alignment is an important feature [7–10]. Cellular alignment is important for the healthy functioning of the organ/tissue (e.g., strong directional muscle contraction), and we believe that our method of aligning cells on biomaterials, simply by applying an electrical current through the conductive biomaterial may facilitate the generation of tissue engineered organ/tissue substitutes with physiologically relevant cell alignment.

## Conclusion & future perspective

Cell alignment within specific tissues is clearly observable within the anisotropically aligned pores observed in bone, cardiac, nerve and other tissues. This has motivated the development of novel materials that instruct the cells thereon/therein to align, most commonly through topological cues engineered into the materials [7–10].

Herein we report the preparation of previously unreported CP-based multilayer films. Films based on poly(3,4-ethylenedioxythiophene) derivatives, chitosan and gelatin were prepared by dip coating and their physicochemical properties characterized. Fibroblasts cultured on their surfaces of such films were shown to adhere and proliferate *in vitro*, and moreover, to respond to a DC current passed through the films by aligning with the current. Such conductive films have prospects for the development of thin conformal bioactive coatings potentially useful for substrates with patterns or more complex topographies than the simple 2D surfaces studied here. Furthermore, the fact that human mesenchymal stem cells adhere to the films (Supplementary Figure 3) highlights their potential for patient specific applications as personalized medical devices for tissue engineering [38–40].

Manufacturing multilayer films by dip coating is very attractive as it is simple and industrially scalable. Moreover, the properties of the films could be easily tuned by altering the contents of the dipping baths. For example, using water soluble CPs with molecular weights below the renal filtration limit of approximately 50 kDa allows the preparation of bioerodible conductive films, and it is likely that adhesion could be improved by coating with mixtures of extracellular matrix-derived proteins (e.g., collagen, fibronectin, laminin). We believe the simplicity of our approach enables us to tailor the properties of the films to specific niche applications (and potentially specific patients).

**Table 1.** **Surface properties of the films.**

**Sample**	**Thickness (μm)**	***Ra* (μm)**	***Rq* (μm)**	**Water contact angle (°)**	**Conductivity σ (S cm^-1^)**
PEDOT-PSS drop cast	3.04 ± 1.67	0.67 ± 0.23	0.99 ± 0.35	51.6 ± 20.3	8.9 × 10^-7^ ± 9.8 × 10^-9^
PEDOT-S drop cast	4.74 ± 1.71	1.71 ± 0.42	2.39 ± 0.58	54.7 ± 5.1	1.8 × 10^-7^ ± 1.5 × 10^-8^
PEDOT-PSS dip-coated	0.57 ± 0.11	0.25 ± 0.04	0.36 ± 0.07	53.1 ± 9.6	3.0 × 10^-4^ ± 4.1 × 10^-5^
PEDOT-S dip-coated	0.15 ± 0.12	0.05 ± 0.02	0.08 ± 0.04	46.6 ± 1.5	3.0 × 10^-3^ ± 5.9 × 10^-4^

Mean results are expressed as mean ± standard deviations.

*Ra*: Average roughness; *Rq*: Root-mean-square roughness.

**Executive summary**

Thin conformable conducting multilayer films of poly(3.4-ethylenedioxythiophene) derivatives, chitosan and gelatin were prepared in a layer-by-layer fashion and cross-linked with genipin.
The conductivity of the films were of a similar order of magnitude to those of mammalian tissues.
Fibroblasts adhered to and proliferated on the films.
Fibroblasts aligned with the dipping direction used during film preparation.
Fibroblast alignment was enhanced by the application of a DC current through the conducting films.
