# Supplementary material for: Conducting polymer-based multilayer films for instructive biomaterial coatings
Source: Future Sci OA. 2015 Nov 2;1(4):FSO79. doi: 10.4155/fso.15.79 (PMC5137882; doi:10.4155/fso.15.79)

**Supplementary information**

**Figure S1.** Experimental setup for dip coating multilayer films using a Gilson 223 Sample Changer converted for use as a dip coater.


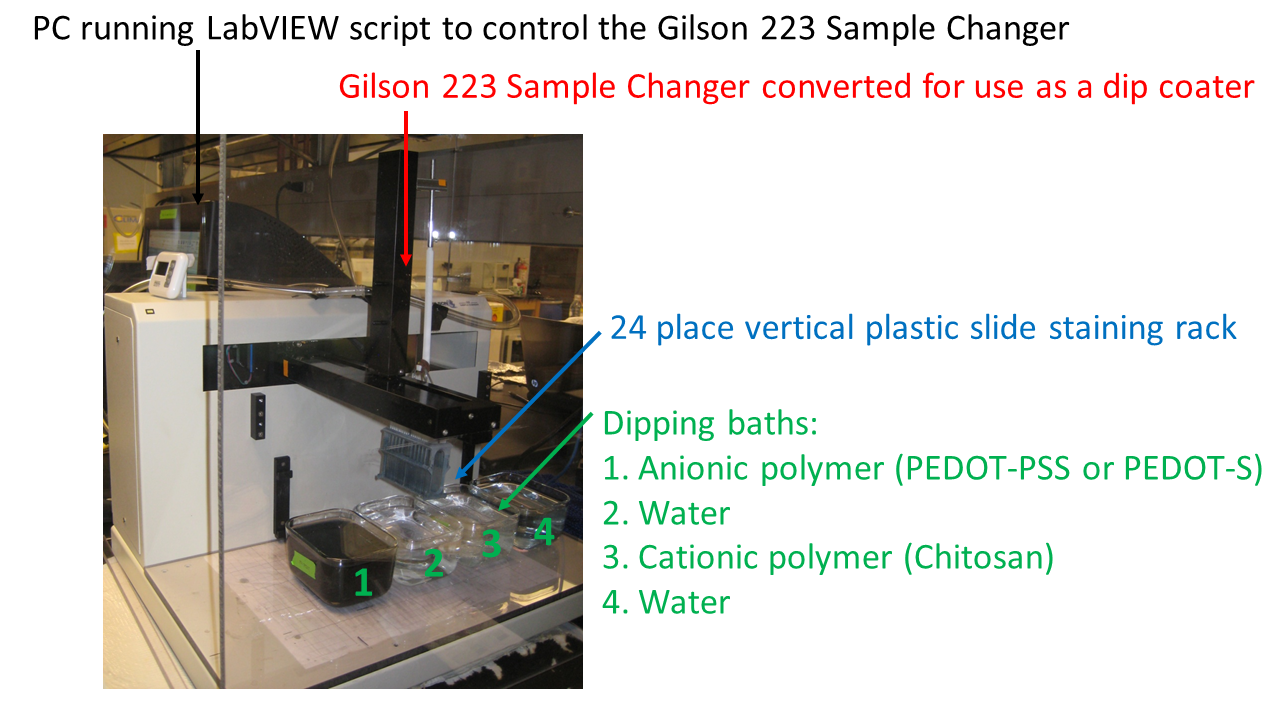


**Figure S2.** Scatter plot correlating the relationship between the cell orientation (angle relative to the dipping direction of the multilayer films) and length of the cells on the polymer films without and with electrical stimulation. A) PEDOT-PSS-based films. B) PEDOT-S-based films. Black circles represent cells without electrical stimulation, whereas red circles represent cells with electrical stimulation; black and red lines represent the corresponding trend lines.

**
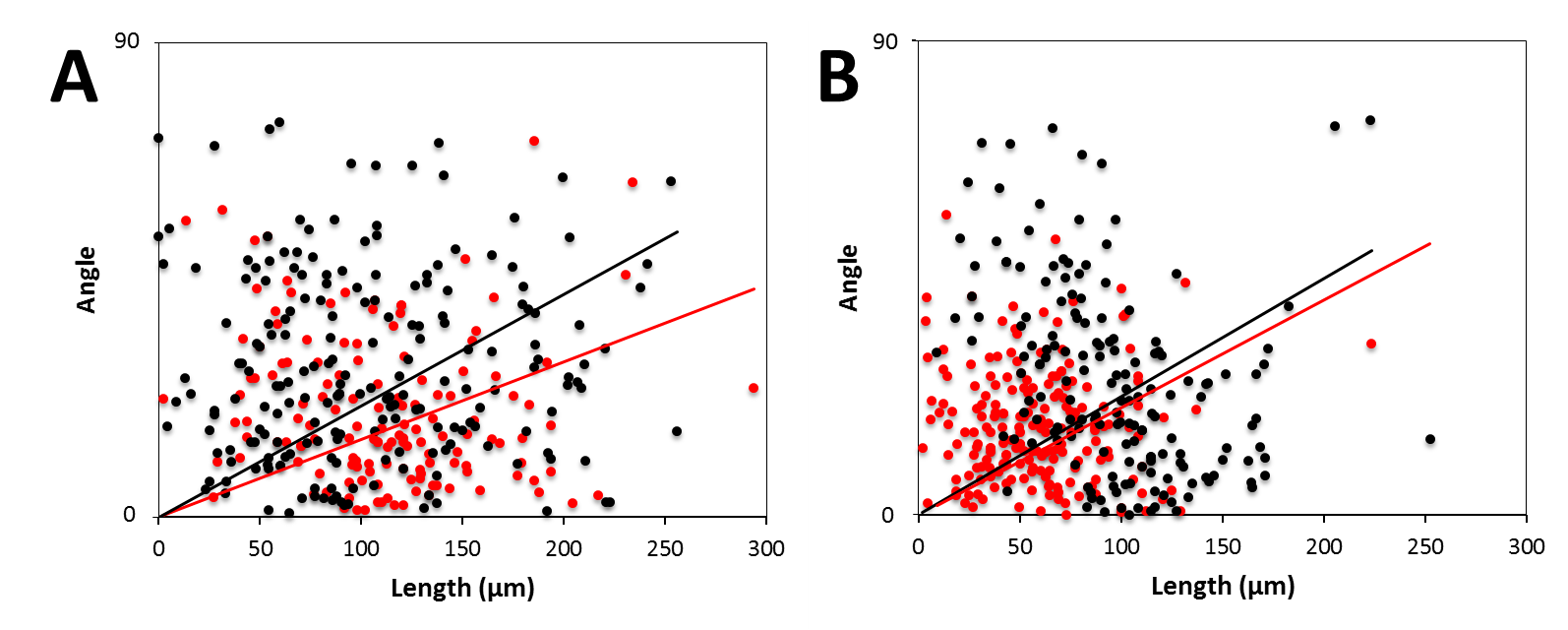
**

Human mesenchymal stem cell adhesion studies

HMSCs were supplied by Lonza (Walkersville, MD). Samples were prepared as described above. After sterilization, the samples were incubated for 30 minutes in 24 well plates containing HMSC growth medium that was composed of: high glucose Dulbecco’s Modified Eagle Medium (DMEM, 440 mL); fetal bovine serum (50 mL); antibiotic-antimycotic (5 mL); non-essential amino acids (5 mL), and 2 ng mL^-1^ basic fibroblast growth factor. Medium was aspirated and replaced prior to HMSC seeding. Cell viability before starting the experiment was determined by the Trypan Blue exclusion method, and the measured viability exceeded 95 % in all cases. HMSCs were seeded at 5,000 cells cm^-2^, and incubated at 37 °C, 95 % humidity, and a CO_2_ content of 5 %. Samples were fixed and stained as decribed above (n = 3).

**Figure S3.** HMSCs on multilayer films after 3 days in culture. A) PEDOT-PSS-based multilayer films. B) PEDOT-S-based multilayer films. DAPI-stained nuclei are blue and Alexa Fluor® 488-stained actin is green. Scale bars represent 150 µm.


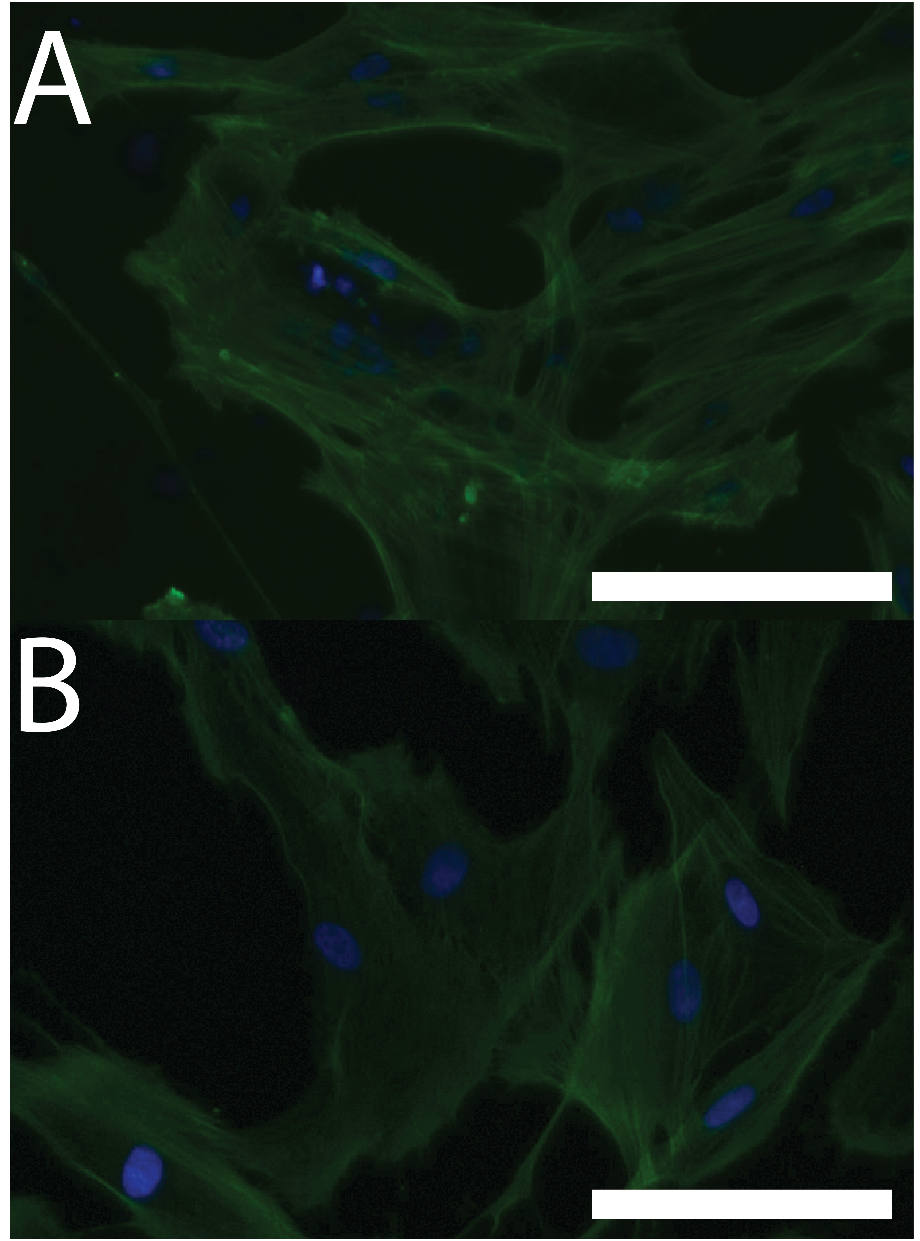

Supplement: Supplementary file 1 [file fso-01-79-s1.docx]
