# Supplementary material for: Conducting polymer-based multilayer films for instructive biomaterial coatings
Source: Future Sci OA. 2015 Nov 2;1(4):FSO79. doi: 10.4155/fso.15.79 (PMC5137882; doi:10.4155/fso.15.79)

Developers: Hetian Li, Austin B. McElroy, John G. Hardy and Christine E. Schmidt
Affiliation: Department of Biomedical Engineering, The University of Texas at Austin

DIP COATER USERS GUIDE

The dip coating system was converted from a Gilson 223 Sample Changer (Gilson, Inc., Middleton, WI, USA). The glass containers used as dipping baths were purchased from the containerstore.com. The slides were held in a 24 place plastic slide staining rack (Fisher Scientific, Austin, TX, USA), typically with one empty slot between each slide. The sample changer was controlled with script written using LabVIEW (National Instruments, Austin, TX, USA).

How to Set Up the Dip Coater

1. Load slide rack with charged glass slides and attach to arm of dip coater.
2. Manually push dip coater arm to root position (X0000/Y0000/Z2150).
3. Turn on dip coater by flipping I/O switch located on back panel.

Figure 1: Experimental Setup

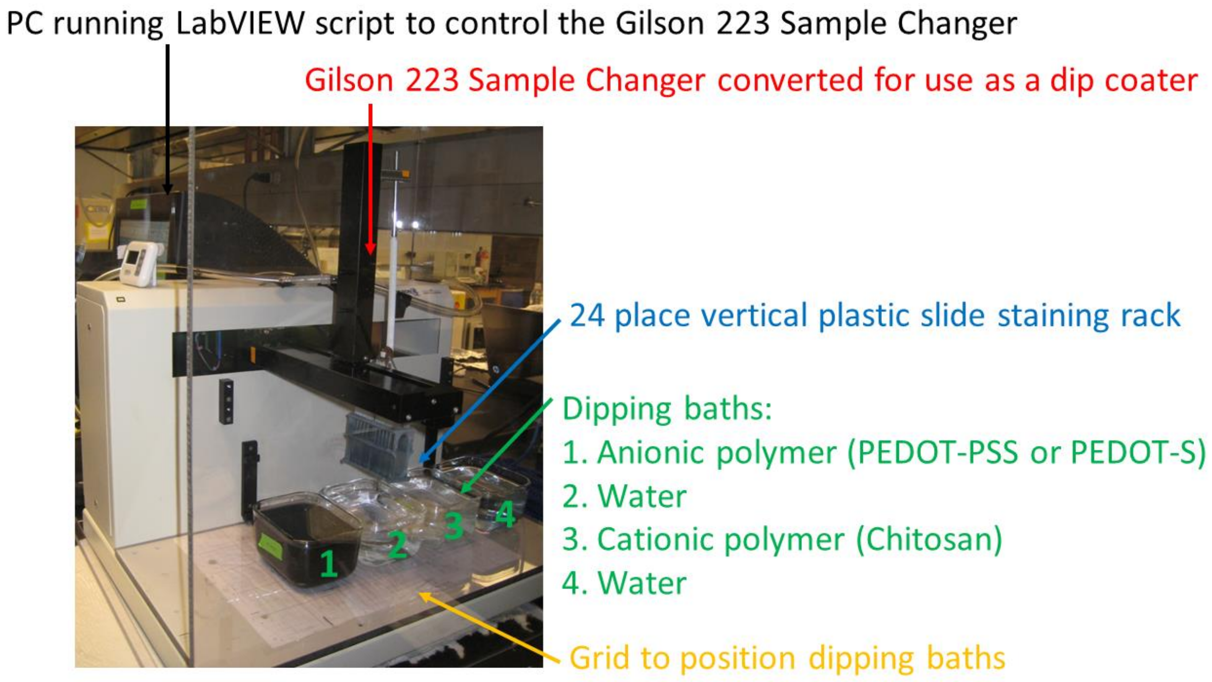

Developers: Hetian Li, Austin B. McElroy, John G. Hardy and Christine E. Schmidt
Affiliation: Department of Biomedical Engineering, The University of Texas at Austin

How to Set Up LabVIEW

4. Open **Gilson 223 Sample Changer.vi** file located on desktop. This is what you should see:

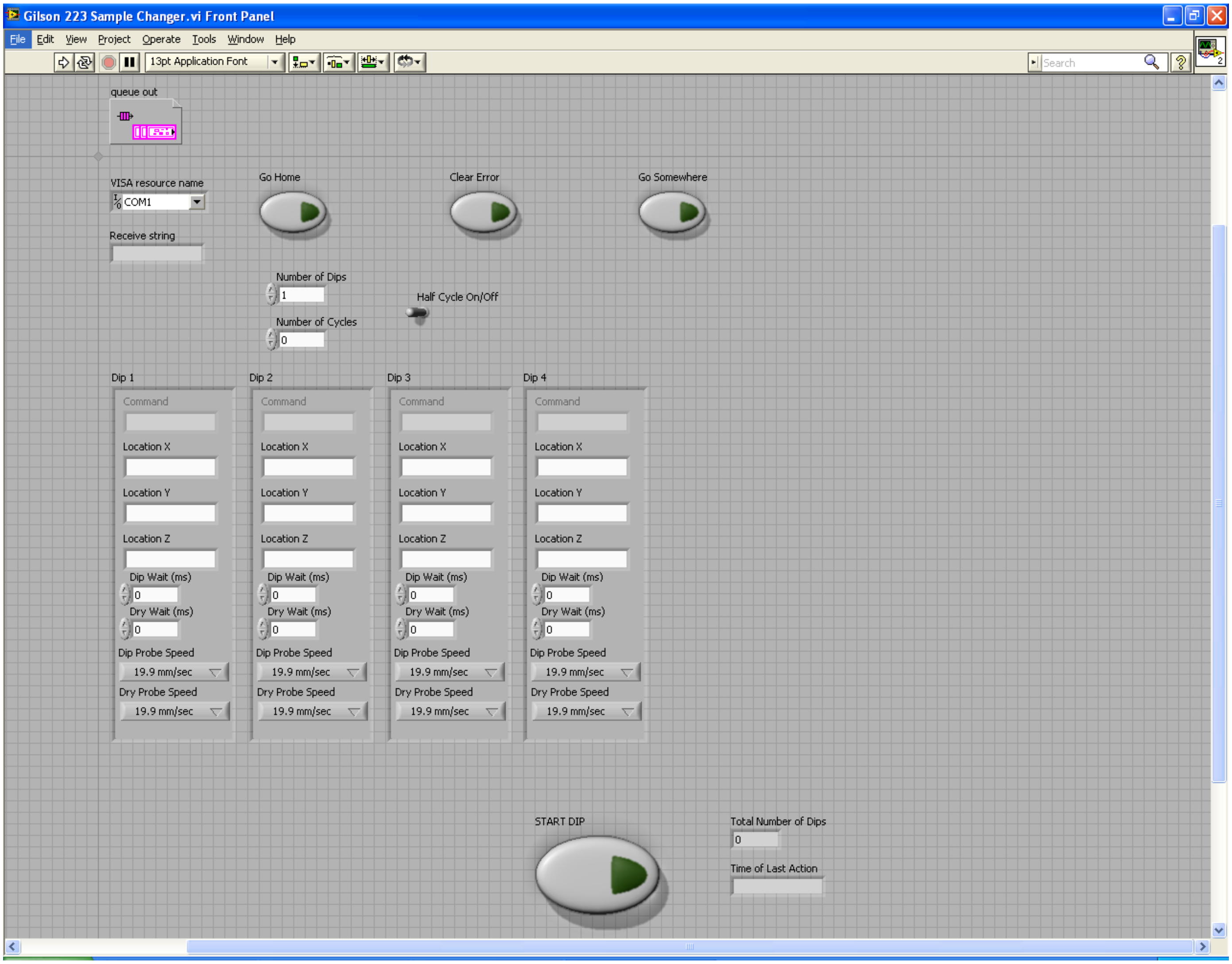

5. Click **Run** icon located on the toolbar.

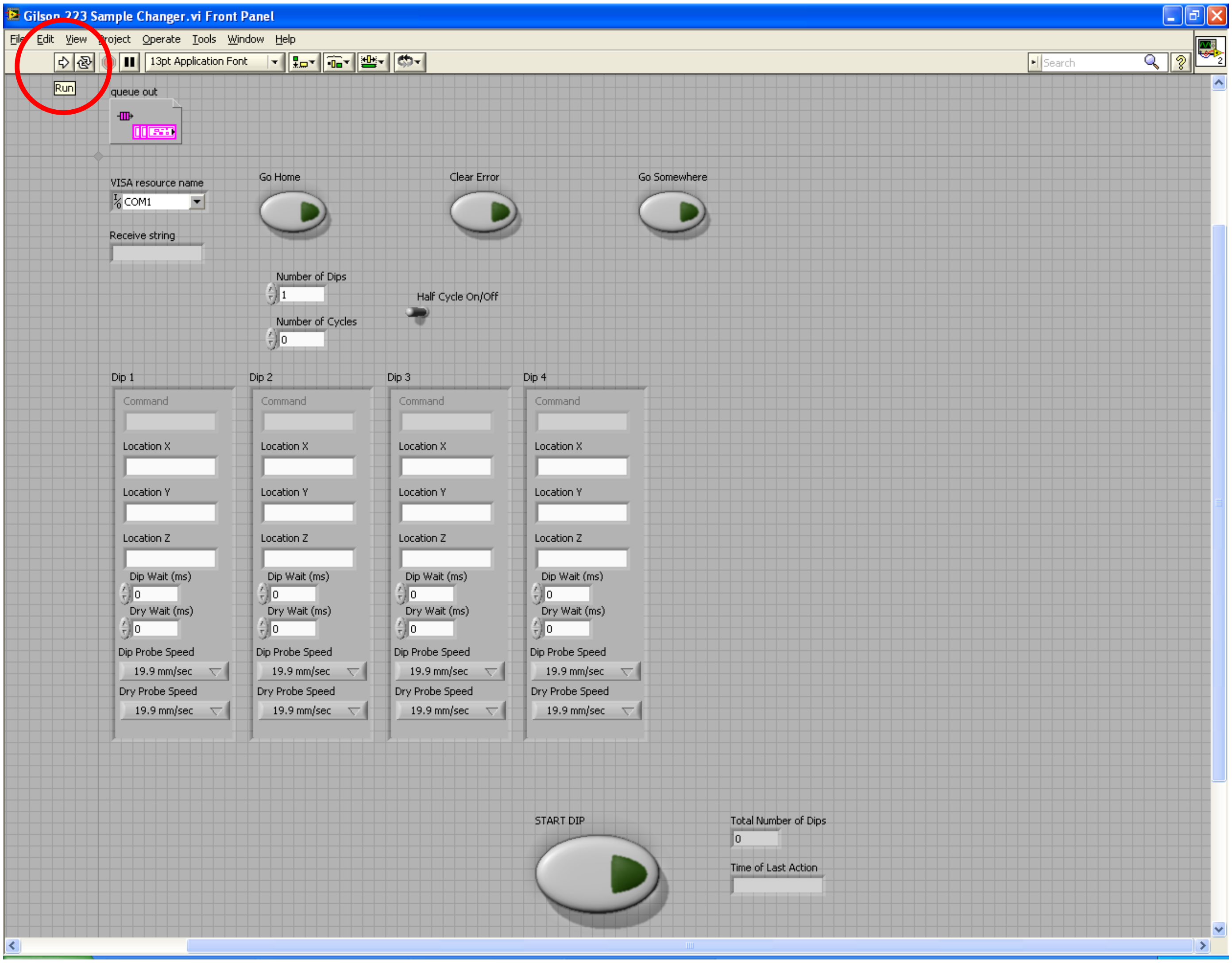

Developers: Hetian Li, Austin B. McElroy, John G. Hardy and Christine E. Schmidt
Affiliation: Department of Biomedical Engineering, The University of Texas at Austin

6. Press **Clear Error** button. This will clear any errors indicated by the blinking LED light on the dip coater. Press **Go Home** button. This will set the dip coater arm to the home position (X0010/Y1200/Z2150).

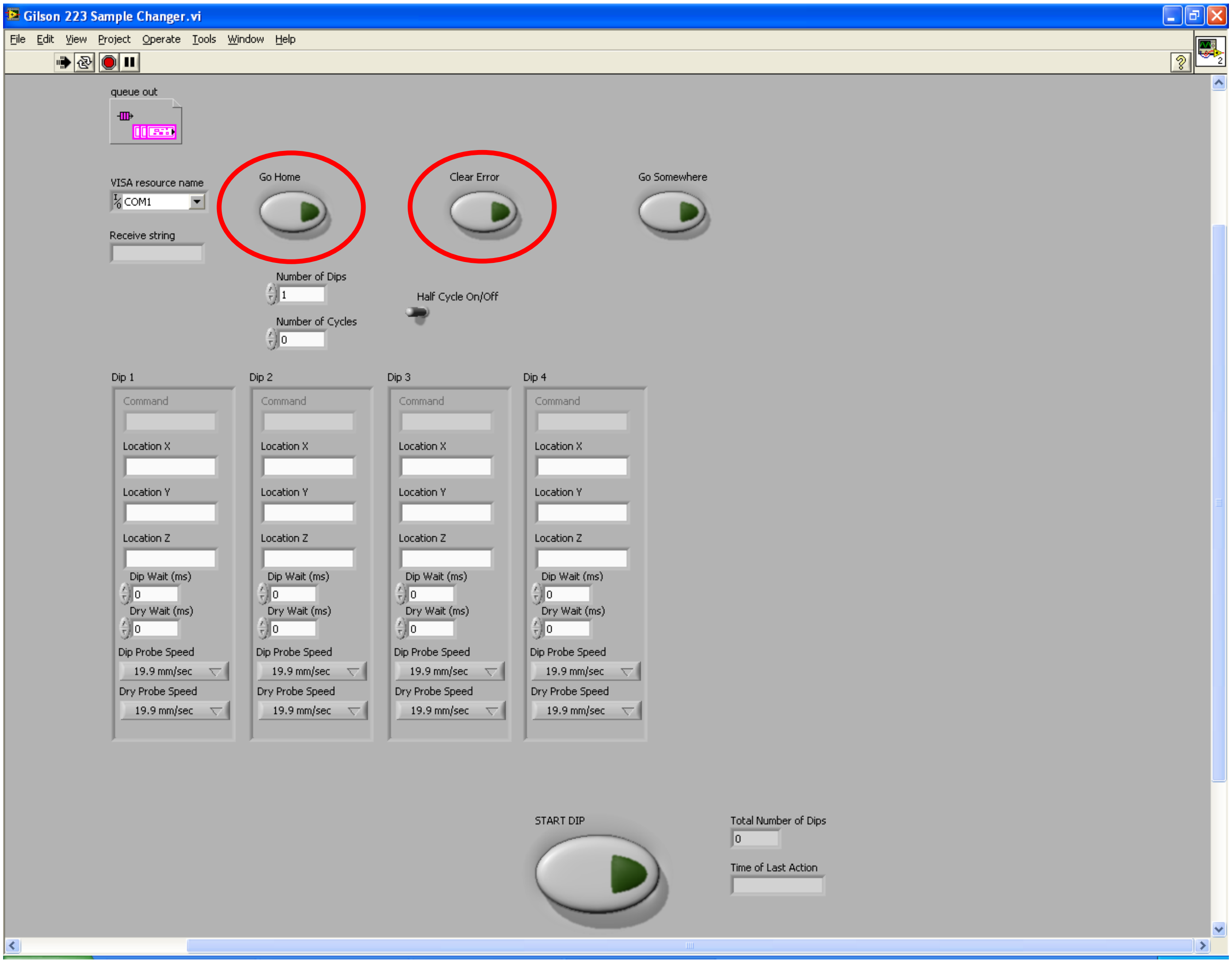

7. Enter the numerical values as follows:

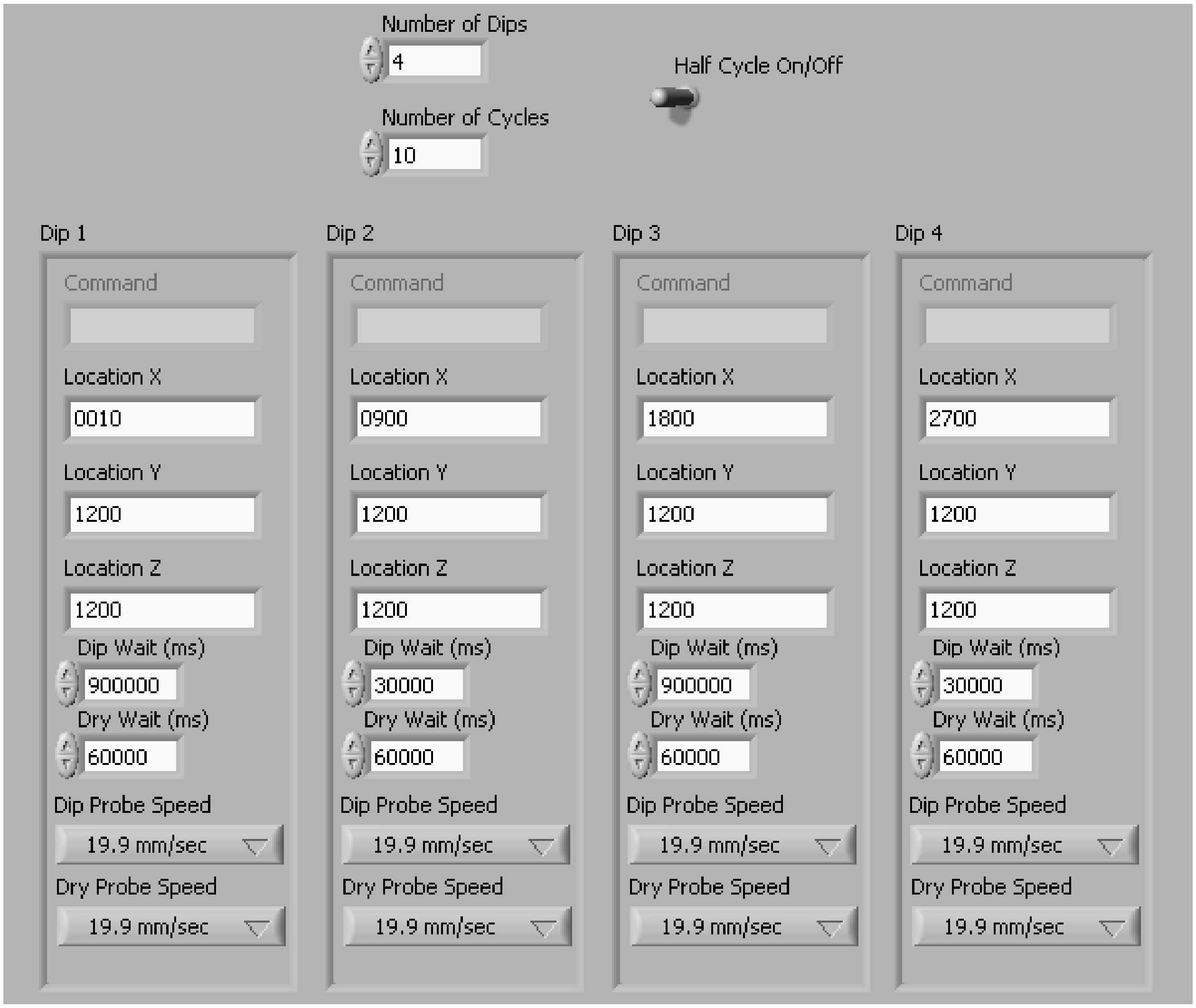

8. Press **START DIP** button. This will begin the dipping process. Each dipping cycle runs for 35 minutes. Ten dipping cycles will run for 350 minutes (5 hours, 50 minutes).

Developers: Hetian Li, Austin B. McElroy, John G. Hardy and Christine E. Schmidt
Affiliation: Department of Biomedical Engineering, The University of Texas at Austin

9. When dipping is complete (Total Number of Dips = 40), click **Abort** icon located on the toolbar and close program. NEVER SAVE.

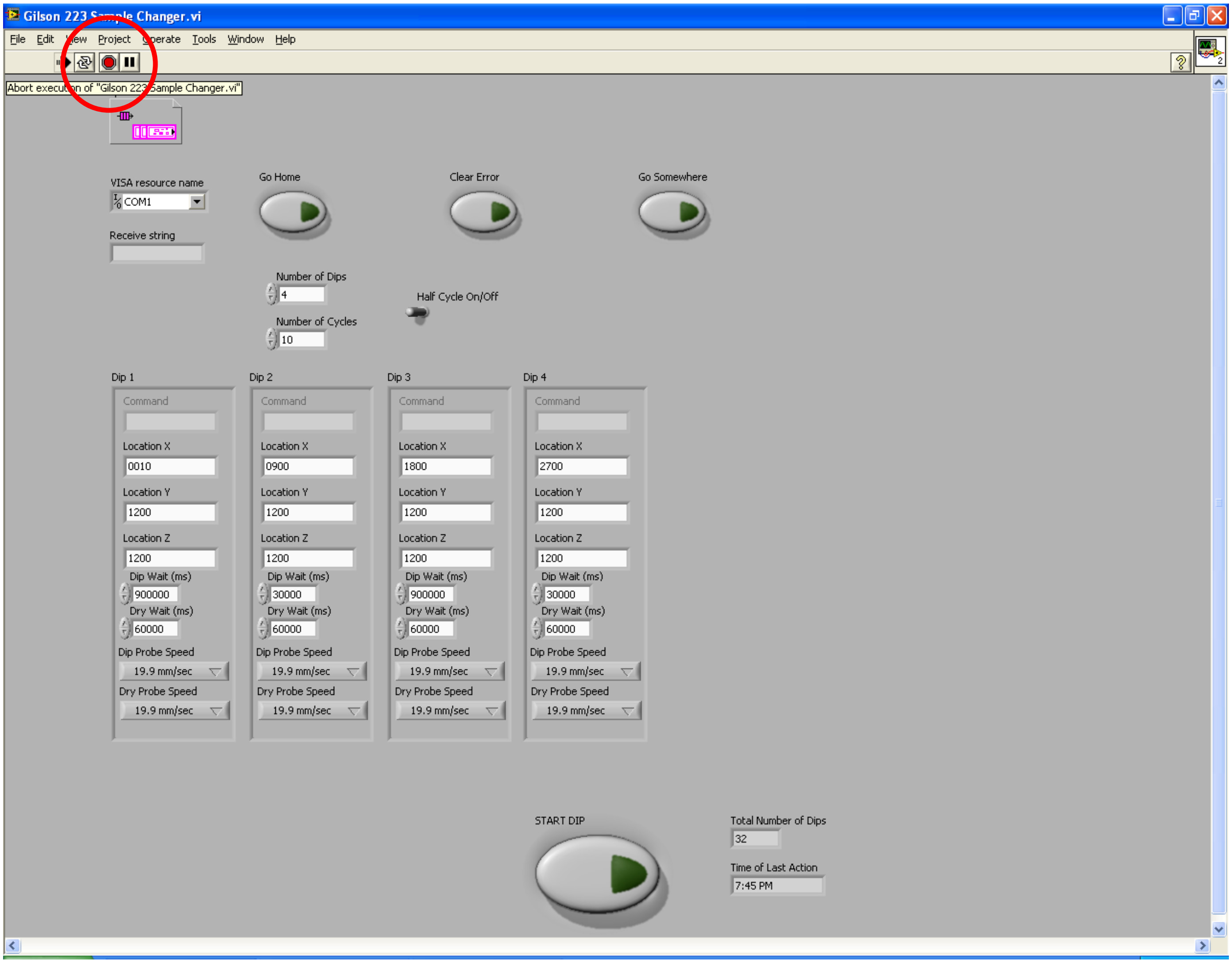

Supplement: Supplementary file 2 [file fso-01-79-s2.zip › labview-script-and-user-guide/Gilson 223 Dip Coater Users Guide.pdf]
