# Supplementary figures and images for: Conducting polymer-based multilayer films for instructive biomaterial coatings
Source: Future Sci OA. 2015 Nov 2;1(4):FSO79. doi: 10.4155/fso.15.79 (PMC5137882; doi:10.4155/fso.15.79)

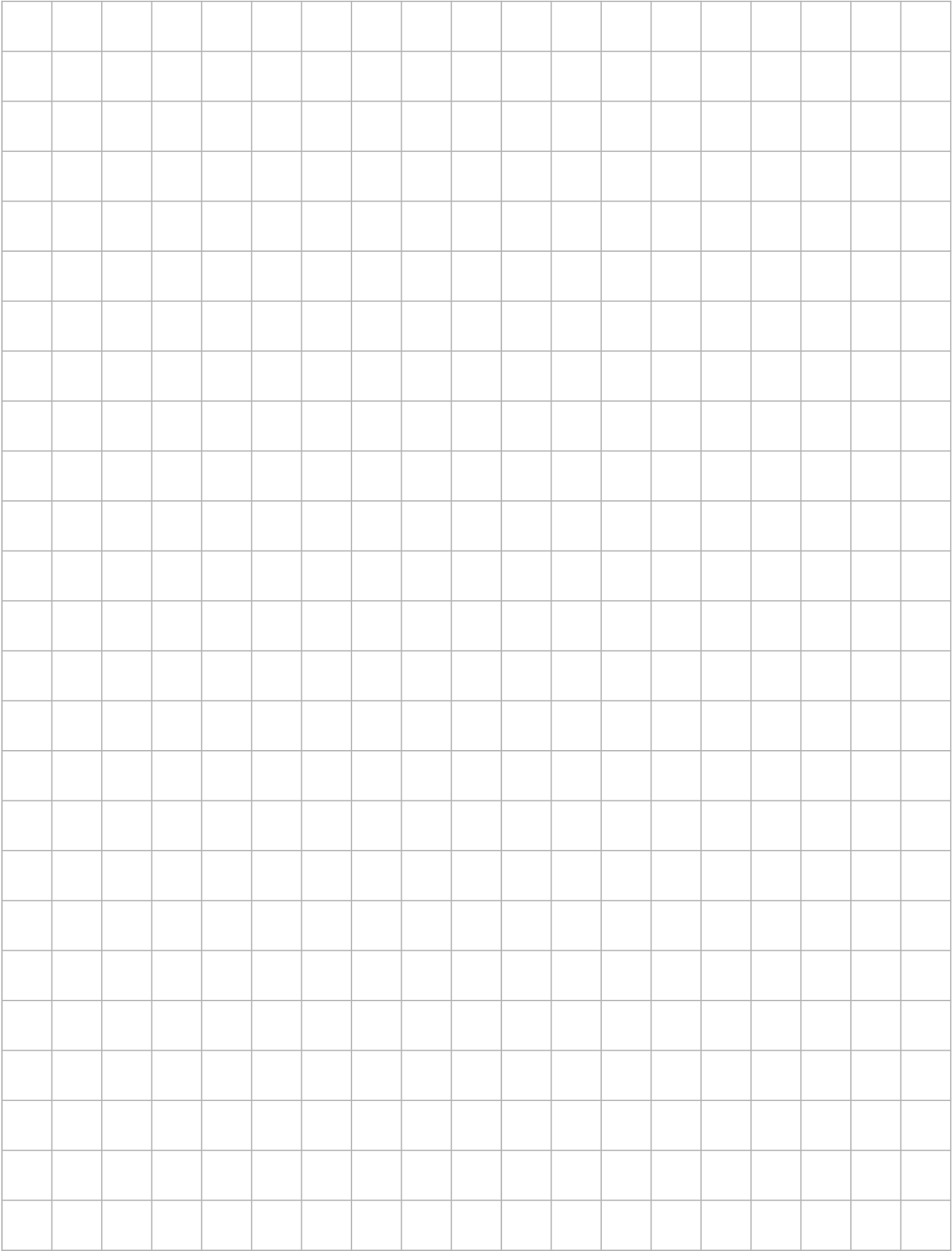

Supplement: Supplementary file 2 [file fso-01-79-s2.zip › labview-script-and-user-guide/Dipcoater-grid-to-position-dipping-baths.pdf]
